# Minimal invasive surgery for unicameral bone cyst using demineralized bone matrix: a case series

**DOI:** 10.1186/1471-2474-13-134

**Published:** 2012-07-29

**Authors:** Hwan Seong Cho, Sung Hwa Seo, So Hyun Park, Jong Hoon Park, Duk Seop Shin, Il Hyung Park

**Affiliations:** 1 Department of Orthopaedic Surgery, Seoul National University College of Medicine, Seoul National University Bundang Hopital, Gyeonggi-do, Korea; 2 Department of Biomedical Sciences, Kyungpook National University, Daegu, Korea; 3 Department of Physical Therapy, Daegu University, Daegu, Korea; 4 Department of Orthopaedic Surgery, Korea University College of Medicine, Seoul, Korea; 5 Department of Orthopaedic Surgery, Yeungnam University College of Medicine, Daegu, Korea; 6 Department of Orthopaedic Surgery, Kyungpook National University School of Medicine, Daegu, Korea

## Abstract

**Background:**

Various treatments for unicameral bone cyst have been proposed. Recent concern focuses on the effectiveness of closed methods. This study evaluated the effectiveness of demineralized bone matrix as a graft material after intramedullary decompression for the treatment of unicameral bone cysts.

**Methods:**

Between October 2008 and June 2010, twenty-five patients with a unicameral bone cyst were treated with intramedullary decompression followed by grafting of demineralized bone matrix. There were 21 males and 4 female patients with mean age of 11.1 years (range, 3–19 years). The proximal metaphysis of the humerus was affected in 12 patients, the proximal femur in five, the calcaneum in three, the distal femur in two, the tibia in two, and the radius in one. There were 17 active cysts and 8 latent cysts. Radiologic change was evaluated according to a modified Neer classification. Time to healing was defined as the period required achieving cortical thickening on the anteroposterior and lateral plain radiographs, as well as consolidation of the cyst. The patients were followed up for mean period of 23.9 months (range, 15–36 months).

**Results:**

Nineteen of 25 cysts had completely consolidated after a single procedure. The mean time to healing was 6.6 months (range, 3–12 months). Four had incomplete healing radiographically but had no clinical symptom with enough cortical thickness to prevent fracture. None of these four cysts needed a second intervention until the last follow-up. Two of 25 patients required a second intervention because of cyst recurrence. All of the two had a radiographical healing of cyst after mean of 10 additional months of follow-up.

**Conclusions:**

A minimal invasive technique including the injection of DBM could serve as an excellent treatment method for unicameral bone cysts.

## Background

Unicameral bone cysts (UBC) are benign fluid-containing lesions lined by a thin membrane. They are found in the metaphysis of long bones with the most common site being the proximal humerus followed by the proximal femur even though they may be found in the calcaneus and pelvis. UBCs occur usually in the first two decades of life. Those cysts that abut a physis are termed active and those separated from the physis are called inactive cysts. UBC may cause repetitive pathological fracture and skeletal deformities during growth because of its proximity to the physis. Surgical procedures for the management have ranged from simple curettage with bone graft [1] to subperiosteal resection [2]. However, considering that the aim of treatment of UBC is the prevention of fracture and skeletal deformity associated with repeated fractures, aggressive open procedures are not justified any more [3-8].

Since the intralesional steroid injection was introduced by Scaglietti in 1974 [9], many percutaneous or minimal invasive procedures have been proposed [10,11]. However, the success rate of percutaneous procedures has been reported to vary [3,12]. Recent studies focus on the effectiveness of percutaneous procedures in terms of recurrence rate and the number of procedures. The basic concept of closed method is decompression of cyst followed by grafting of osteogenic materials. Many authors have tried injecting different graft materials. Demineralized bone matrix (DBM) has recently served as a bone substitute in many orthopedic fields and favorable results were reported [7,13,14].

The purpose of this study was to evaluate the effectiveness of DBM as a graft material after intramedullary decompression for the treatment of unicameral bone cysts in terms of the overall success rate, recurrence and the time to healing.

## Methods

Between October 2008 and June 2010, twenty-five patients who were diagnosed with unicameral bone cyst were enrolled in this study. There were 21 boys and 4 girls, with a mean age of 11.4 years (range, 3–19 years). The mean follow-up period was 23.9 months (range, 15–36 months). The proximal metaphysis of the humerus was affected in 12 patients (48%), the proximal femur in five (20%), the calcaneum in three (12%), the distal femur in two (8%), the tibia in two (8%), and the radius in one (4%). There were 17 active cysts and 8 latent cysts. In all patients, the diagnosis was made after radiographic workup for pain with activities of daily living or secondary to a pathologic fracture. Eight patients (32%) had experienced more than one pathologic fracture before being admitted to our clinic. For the patients with recent pathologic fracture, management for UBC was postponed at least 3 months because obliteration of cyst after healing of fracture may occur although it is not the rule. In this study, all fractures were managed conservatively and no patient achieved obliteration of cyst after healing of fracture.

All procedures were performed by the senior authors (IH Park, DS Shin) under general anesthesia and guided by fluoroscopy. A less than 2-centimeter-skin incision was made over the thinnest accessible wall of the cyst avoiding neurovascular structures. A cortical window was created to allow sweeping movement of curettes and moving along the cyst lining. A sample tissue was sent to histopathological analysis; diagnosis of UBC was confirmed in all patients. The content of the cyst was aspirated and intramedullary decompression was performed with a curette. Any septa within the cysts were also removed by curettes. After washing out the cyst with normal saline, gel-type DBM (ExFuse™, Hansbiomed Inc, Seoul, Korea) was injected. The volume of the DBM was determined by the size of the cyst, as measured on the radiographs, using the formula (length × width × height × π/6) [15]. The mean volume injected was 12 milliliters (range, 5–25 milliliters). The wound was closed at least 10 minutes after injection of DBM to consolidate and minimize the leakage of DBM. No metallic implant was employed for stability in any patient. All patients were discharged the day after surgery.

Physical activity was restricted for four weeks in patients with a lesion in the upper extremity and six weeks in those with a lesion in the lower extremity. Radiographs were taken every month after the procedure for three months after surgery and repeated every three months until evidence of healing was observed. Radiologic change was evaluated by three musculoskeletal radiologists according to a modified Neer classification [1] (Table 1). Time to healing was defined as the period required achieving cortical thickening on the anteroposterior and lateral plain radiographs, as well as consolidation of the cyst. Interobserver agreement was determined by Fleiss’ kappa test [16], which is a statistical measure for assessing the reliability of agreement between more than 2 observers. This study was approved by the institutional review board at Kyungpook national university hospital (KNUHMD_07-0005). All patients included in this study were informed that their cases would be submitted for publication, and consented.

**Table 1:** Modified Neer classification of radiological results

**Classification**	**Description**
Healed	Cyst filled by new bone, with or without small radiolucent area(s) < 1 cm in size
Healing with defects	Radiolucent area(s) < 50% of the diameter of bone, with enough cortical thickness to prevent fracture
Persistent cyst	Radiolucent area > 50% of diameter of the bone and with a thin cortical rim. No increase in the size of the cyst. Continued restriction of activity or repeated treatment is required
Recurrent cyst	Cyst reappeared in a previously obliterated area, or a residual radiolucent area has increased in size

## Results

The details of the 25 patients are given in Table 2.

**Table 2:** Details of patients

**No.**	**Gender**	**Age (yrs)**	**Location**	**Active/Latent**	**Size (mm**^**3**^**)**	**Previous fracture (Yes/No)**	**Time to healing (months)**	**Status**
1	M	5	Proximal femur	Active	39 × 31 × 29	Yes	3	Recurrent cyst*
2	M	12	Calcaneus	Latent	20 × 20 × 29	No	4	Healed
3	M	12	Distal femur	Active	25 × 16 × 25	No	4	Healed
4	M	6	Proximal humerus	Active	72 × 26 × 20	No	4	Healed
5	M	16	Proximal humerus	Latent	69 × 30 × 25	Yes	8	Healed
6	M	13	Proximal humerus	Active	39 × 20 × 20	Yes	6	Healed
7	F	12	Proximal humerus	Active	18 × 15 × 20	No	8	Healed
8	M	10	Proximal humerus	Active	37 × 16 × 15	No	6	Healed
9	M	14	Proximal humerus	Latent	35 × 27 × 25	Yes	10	Healed
10	F	10	Proximal humerus	Active	60 × 18 × 16	No	6	Healed
11	M	10	Proximal humerus	Active	70 × 25 × 25	No	6	Healed
12	F	10	Distal femur	Active	75 × 30 × 23	No	6	Healed
13	M	7	Distal radius	Active	11 × 12 × 9	Yes	8	Healing with defect
14	M	12	Proximal humerus	Active	25 × 28 × 20	Yes	12	Healed
15	M	10	Proximal humerus	Active	36 × 17 × 15	No	8	Healing with defect
16	M	3	Proximal humerus	Active	35 × 17 × 28	Yes	3	Recurrent cyst*
17	M	11	Proximal femur	Active	35 × 20 × 20	No	10	Healing with defect
18	M	19	Proximal femur	Latent	60 × 25 × 25	No	10	Healed
19	M	8	Distal tibia	Latent	18 × 14 × 14	No	6	Healing with defect
20	M	18	Proximal femur	Active	50 × 15 × 20	Yes	9	Healed
21	M	13	Proximal femur	Active	25 × 20 × 13	No	6	Healed
22	M	18	Middle humerus	Active	36 × 15 × 13	No	7	Healed
23	M	8	Calcaneus	Latent	18 × 18 × 15	No	4	Healed
24	M	10	Calcaneus	Latent	18 × 13 × 15	No	5	Healed
25	F	11	Proximal tibia	Latent	40 × 13 × 15	No	7	Healed

* It was difficult to define time to healing for recurrent cases. Consolidation of DBM within cyst was considered as initial time to healing regardless persistent cyst.

**Figure 1:**
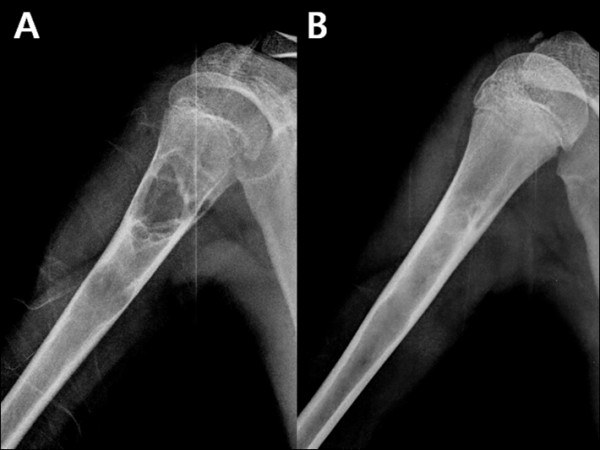
**Radiographical cyst healing after injection of DBM.** Plain radiographs **A**) of the right humerus before surgery in a 13-year-old boy with cystic lesion located at the proximal meta-diaphysis **B**) at postoperative 24 months.

**Figure 2:**
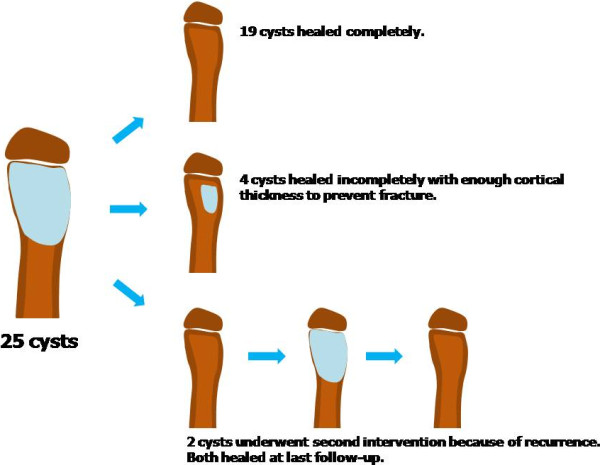
Serial diagrams of final result of 25 cysts.

The permanent pathology report confirmed all cysts as UBC. At 4–6 weeks postoperatively, all patients were pain free and had full range of motion of the adjacent joint. Full activity including weight-bearing was resumed within this time in all patients. At the last follow-up, all patients were asymptomatic. Radiographical cyst healing in terms of cortical remodeling was seen at a mean of 6.6 month follow-up (range, 3–12 months) (Figure 1). Fleiss’ kappa value on rating of the modified Neer classication was 0.763. Two patients required a second intervention because of cyst recurrence. The mean interval from initial intervention to a second was 9.0 months (range, 4–14 months). Both recurrences were active cysts located in the proximal humerus and proximal femur. Both patients had a radiographical healing of cyst after mean of 10 additional months of follow-up. Four of the 25 patients had incomplete healing radiographically showing small, persistent radiolucent areas within the original cyst but had enough cortical thickness to prevent fracture. None of four patients needed second intervention until the last follow-up (Figure 2).

**Figure 3:**
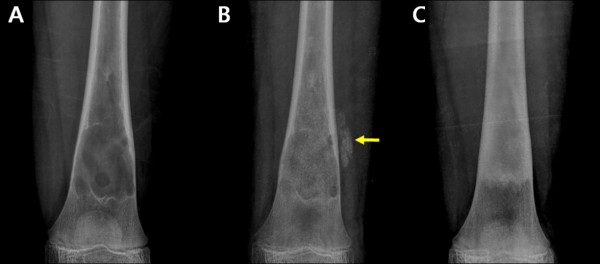
**Leakage of DBM.** Plain radiographs **A**) of the distal femur in a 10-year-old girl **B**) at postoperative one month showing leakage of DBM (arrow) **C**) at the postoperative 13 months showing complete resorption of extraosseous DBM.

Extraosseous mineralization owing to leakage of DBM was found in 2 patients, no additional surgery was necessary associated to DBM leakage. The leakage DBM completely resorbed within 3 months in both cases (Figure 3). There were no other significant complications related to the procedure, nor did any fracture occur after initiation of the above regimen.

## Discussion

Although there is no standardized treatment for UBC, the goal of treatment is to prevent pathological fracture and skeletal deformities during growth associated to repeated pathological fractures [3]. A painful lesion with precarious cortical thinning demands surgical intervention, rendering the bone at risk for pathological fracture. Surgical procedures include injection of corticosteroid [12,17,18] or bone marrow [11,18], decompression with cannulated screw(s) [6], intramedullary nailing [19,20] and open curettage followed by bone grafting [21-23]. In this study, we evaluated the effectiveness of DBM as a graft material in UBC treatment in terms of overall healing rate, recurrence, time to healing and associated complication. In the present study, the healing rate with a single intervention was 92% (23/25) and it reached 100% after a second procedure.

To explain the etiology of UBC, several theories have been proposed including a true intra-osseous synovial cyst [24], the degenerative phase of a benign tumor [25], failure to resorb hematomas [26], low-grade form of osteomyelitis [27], and venous obstruction [28]. Of these theories, venous obstruction has been suggested as being the most probable cause of UBC. Considering that venous obstruction model is preferred, decompression of cyst and injection of steroid or osteogenic materials has replaced aggressive open procedure and bone grafting [3,7,8]. There have been many reports on minimal invasive procedures for the treatment of UBC [12,17,18,29]. Scaglietti [9] first described the percutaneous injection of methylprednisolone acetate as a treatment of UBC in 1974. Subsequently, many authors have reported satisfactory result with high success rates ranging from 50% to 90% [21,22,29,30] Simplicity and low morbidity associated with steroid injection made it popular. However, usually several procedures are necessary to achieve consolidation of the cyst. The classic report by Scaglietti using percutaneous steroid injection showed only 24% healing rate after a single injection [18]. Many other studies also suggested that close radiological surveillance should be maintained and repeated steroid injections may be needed to achieve adequate consolidation [12,17,18]. Steroid injection might inhibit production of bone resorptive factors by its anti-inflammatory effect. In addition, it reduces internal pressure of cyst through trepanation. However, steroid injection does not provide bone-forming potential in itself.

**Figure 4:**
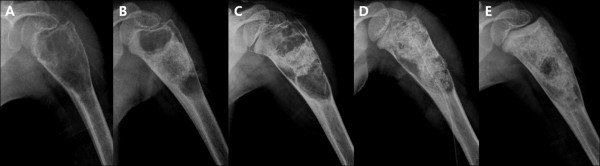
**Insufficient DBM in quantity leaded to inadequate healing of the cyst.** Plain radiographs **A**) of the proximal humerus in a 3-year-old boy **B**) at postoperative 3 months showing filling defect of DBM **C**) at postoperative 14 month showing recurrence of cyst **D**) after repeat intervention **E**) at 13 additional months of follow-up after a second intervention.

Since autologous bone marrow grafting for UBC treatment was introduced by Lokiec in 1996 [31], the injection of bone marrow alone or in combination with DBM has been proposed as an alternative to steroid injection. The injection of bone marrow provides osteoprogenitor cells and DBM could stimulate new bone formation owing to its osteoinductive and osteoconductive properties [5,14,32]. Recently, many authors have evaluated the effectiveness of DBM as a graft substitute and it is applied in many surgical grafting procedures including spinal fusion, joint reconstructive surgery, trauma and oral/maxillofacial surgery. With respect to the use of DBM in UBC treatment, the effectiveness as a graft material after intramedullary decompression has been evaluated by some authors [7,10,11,29,33] Kanellopoulos et al [13] and Rougraff et al [14] reported about 90% success rate after a single procedure using a mixture of DBM and autologous bone marrow and it took six to nine months to achieve cortical remodeling radiographically. In the present study, we had a cumulative success rate of 100%. In addition, the mean time to healing was 6.6 months. In the current study, two cysts required a repeat procedure. In both cases, the amount of DBM injected was not enough to fill the entire cyst (Figure 4). The basic concept of percutaneous procedure is the intramedullary decompression followed by grafting of osteogenic materials. The importance of induction of osteogenesis following intramedullary decompression in UBC treatment has been advocated by several authors. The high recurrence rate of steroid injection was probably caused by absence of osteogenic potential. In this regard, DBM could be a good grafting substitute for UBC treatment after intramedullary decompression owing to its excellent osteogenic property. In addition, it would be desirable to use an adequate amount of DBM to fill the entire cyst. In the case of Figure 3, it seemed that the insufficient DBM in quantity led to inadequate healing of the cyst and eventually cyst recurrence.

ExFuse™ (Hansbiomed Inc, Seoul, Korea) used in the current study is a mixture of carboxyl methyl cellulose carrier and DBM extracted from freeze-dried allograft with preserving its osteoinductive and osteoconductive properties.

In the present study, we made a small skin incision and created a cortical window over the thinnest accessible wall of the cyst. This made it possible to allow passage and sweeping movement of curettes and made it easy to decompress the intramedullary pressure, remove the cyst lining and get biopsy material. Even though our procedure may seem to be a more aggressive than other percutaneous procedures, the high success rate in the present study may be attributed to more aggressive removal of cyst lining and intramedullary decompression through a cortical window Killian [8]. Another advantage of making a cortical window includes obtaining the biopsy material. Some malignant lesions such as Ewing’s sarcoma or osteosarcoma may show cystic features radiologically [34,35]. Therefore, obtaining biopsy material is important to confirm the diagnosis of UBC.

Our study has several limitations. First, we could not perform any sound analysis to find associated prognostic factors for persistent cyst or recurrence because the number of patients enrolled was relatively small. However, the recurrence rate of the cyst was low (8%) even though this study included many active cysts in younger patients, which are known to be aggressive and resistant to treatment [19,36]. Another limitation of our study is the absence of a comparative group or randomized comparison. In future study, large scale and long-term follow-up clinical studies are required.

## Conclusions

Minimal invasive technique followed by DBM grafting could serve as an excellent treatment method for UBC. The method offers low morbidity, a short hospital stay, and a high success rate. In addition, the advantage of making a small cortical window allows easy decompression of cyst, complete removal of cyst lining and obtaining biopsy material.

## Competing interests

The authors declare that they have no competing interests.

## Authors’ contributions

All authors read and approved the final manuscript.

## Pre-publication history

The pre-publication history for this paper can be accessed here:

http://www.biomedcentral.com/1471-2474/13/134/prepub
